# Real-life effectiveness and safety of salbutamol Steri-Neb™ vs. Ventolin Nebules^®^ for exacerbations in patients with COPD: Historical cohort study

**DOI:** 10.1371/journal.pone.0191404

**Published:** 2018-01-24

**Authors:** David B. Price, Eran Gefen, Gokul Gopalan, Rosie McDonald, Vicky Thomas, Simon Wan Yau Ming, Emily Davis

**Affiliations:** 1 Centre of Academic Primary Care, Division of Applied Health Sciences, University of Aberdeen, Aberdeen, United Kingdom; 2 Observational & Pragmatic Research Institute, Singapore, Singapore; 3 Teva Pharmaceuticals, Petach Tikva, Israel; 4 Teva Pharmaceuticals, Frazer, Pennsylvania, United States of America; Lee Kong Chian School of Medicine, SINGAPORE

## Abstract

**Introduction:**

Ventolin Nebules^®^ (reference product; GlaxoSmithKline) was the first licensed nebulizer solution containing the rapid-onset, short-acting β_2_-agonist salbutamol. Salbutamol Steri-Neb^™^ (comparator; Teva Pharmaceuticals, Inc.) has the same chemical composition as the reference product. This study evaluated whether the effectiveness of the comparator is non-inferior to the reference product alongside concomitant medications during real-life clinical management of COPD exacerbations. Safety in terms of adverse events (AEs) was also examined.

**Methods:**

This matched (1:1) historical cohort study evaluated data from 2 UK primary care databases on patients prescribed the salbutamol comparator or reference. The study included a 1-year baseline period, starting 1 year before the index prescription date, and 1-year outcome period. Cohorts were matched for baseline COPD respiratory medications. The primary outcome was analysis of non-inferiority for the comparator versus reference product for the rate of moderate and severe COPD exacerbations. Non-inferiority was satisfied if the 95% confidence interval (CI) upper limit for mean differences in proportions between treatments was <15%. Secondary outcomes were examined through rate ratios (RR) of severe exacerbations and AEs.

**Results:**

After matching, 1191 patients were included in each cohort. Adjusted upper 95% CI for the difference in proportion of patients experiencing moderate or severe exacerbations between comparator and reference groups was 0.032 (3.2%), demonstrating non-inferiority. No significant differences were observed in rates of moderate and severe exacerbations (RR: 1.00; 95% CI: 0.91, 1.10), severe exacerbations (RR: 0.76; 95% CI: 0.49, 1.17), or AEs (RR: 0.98; 95% CI: 0.78, 1.22) after adjusting for baseline confounders. No significant differences across cohorts were observed for rates of any AE or death.

**Conclusion:**

This matched cohort study of real-life management of COPD patients supports the salbutamol comparator as non-inferior to the reference product, providing an effective treatment alternative for COPD exacerbations. Comparator and reference safety profiles were similar.

## Introduction

Chronic obstructive pulmonary disease (COPD) is a common, progressive, inflammatory disease characterized by persistent airflow limitation [1]. It is a significant cause of morbidity and is predicted to be the third leading cause of death worldwide by the year 2030 [1,2].

Acute worsening of respiratory symptoms requiring a change in treatment, known as exacerbations, often occur in COPD patients. Exacerbations can be triggered by numerous factors, such as respiratory infections or environmental pollutants, and contribute to the overall severity of an individual patient’s disease [1]. Exacerbations are also associated with an accelerated decline in lung function and significant mortality [1,3]. For these reasons, the Global Initiative for Chronic Obstructive Lung Disease (GOLD) guidelines and guidelines from organizations such as the American College of Chest Physicians and the Canadian Thoracic Society recommend that treatment of stable COPD include strategies to prevent and treat exacerbations [1,3,4].

Short-acting β_2_-agonists (SABAs) represent an important treatment option for managing stable COPD and for treating COPD exacerbations. Consistent with guidelines that recommend their use as an alternative add-on therapy for symptom relief in patients with moderate-to-severe COPD and for the treatment of COPD exacerbations [1,3], SABAs are commonly prescribed for treatment of acute exacerbations [5]. Importantly, the effective treatment of an exacerbation serves to minimize its impact on lung function decompensation and is critical to prevent hospitalizations and prevent subsequent relapses with exacerbations [3,6]. Progressively increased risk for subsequent severe exacerbations following a second severe exacerbation has been shown [7]. SABAs with or without short-acting anticholinergics are typically the preferred bronchodilators to effectively treat current exacerbation, thereby helping to prevent subsequent exacerbations [3].

Ventolin Nebules^®^ (GlaxoSmithKline), the reference product in this study, was the first nebulizer solution containing the rapid-onset SABA salbutamol, which was marketed worldwide for bronchospasm relief in patients with COPD [8]. Salbutamol Steri-Neb^™^ (Teva Pharmaceuticals, Inc.), the comparator, is a generic version of the reference product with the same chemical composition as the reference product and both treatments are indicated in Europe for management of chronic bronchospasms and severe acute asthma attacks [8,9].

Costs associated with COPD and its treatment have significantly increased in recent years [10–12]. The cost of managing exacerbations is particularly substantial. In fact, recent estimates suggest that annual healthcare costs are 10 times higher for patients with COPD with acute exacerbations compared with those without them [11]. Because the cost of medications is a substantial contributor to recent increases in COPD costs [10], affordable pharmacologic alternatives for treating and preventing exacerbations are needed.

The primary aim of the present study was to evaluate whether the effectiveness of the salbutamol comparator is non-inferior to that of the reference product in a broad population of COPD patients, alongside possible concomitant respiratory therapies during real-life clinical management of COPD. We also compared the safety profile of the 2 treaments. We used a historic cohort study design to evaluate real-life outcomes in patients with COPD in the UK, where the salbutamol comparator has been used since 1992.

## Methods

### Study design

We conducted a historical, matched-cohort study of patients with COPD treated in primary care practices in the UK using information from 2 databases: the Optimum Patient Care Research Database (OPCRD [13]) and the Clinical Practice Research Datalink (CPRD [14]). The OPCRD provides anonymous patient information collected from 430 general practices in the UK, including data on demographics, investigations, diagnoses, treatment and treatment outcomes, patient-reported outcomes, and events leading to withdrawal of treatment. We supplemented OPCRD data with data from the CPRD database to ensure that appropriate numbers of patients were included in the study. The CPRD database contains anonymous longitudinal data from approximately 650 UK primary care practices.

This study included a 1-year baseline period preceding the index prescription date, and a 1-year outcome period after the index prescription date (S1 Fig). The index prescription date was defined as follows: for patients with COPD who were not on SABA nebulizers at baseline, the index prescription date was the date of first prescription for either the reference product (salbutamol sulfate for inhalation via nebulizer in single-dose ampules of 2.5 mg/mL or 5.0 mg/2.5 mL) or the comparator (salbutamol sulfate for inhalation via nebulizer in single-dose ampules of 2.5 mg/mL or 5.0 mg/2.5 mL) (initiation subcohort); for patients on the reference product at baseline, the index prescription date was the date of first prescription for the comparator (change subcohort) or of first prescription to continue treatment with the reference product (continuation subcohort). Two cohorts of patients were then defined as follows: the comparator cohort, including patients who initiated on or changed to the comparator, and the reference cohort, including patients who initiated on or continued using the reference product. In order to ensure an adequate sample size in each cohort, patients within the change and continuation subcohorts with different prescription dates may have been included more than once if they satisfied the inclusion/exclusion criteria (described below). However, during the matching process, we ensured that only unique patients were analyzed.

In the analysis, we included data that were collected in a period beginning 1 year before the launch of the salbutamol comparator in the UK (May 1991) and continuing through the date of last available data in the OPCRD (February 28, 2013) and CPRD (December 21, 2009). The outcome period was used to compare drug effectiveness and safety between cohorts.

### Ethical approval

The OPCRD has been approved by the Trent Multicentre Research Ethics Committee for use in clinical research. Its governance is provided by the Anonymised Data Ethics Protocols and Transparency (ADEPT) Committee, which is the independent scientific advisory committee for the OPCRD. The study was designed, implemented, and registered in accordance with the criteria of the European Network of Centres for Pharmacoepidemiology and Pharmacovigilance (ENCEPP/SDPP/7645). Informed consent was not needed as patient information was anonymous.

### Patient population

Patients were eligible for inclusion in the study if they met the following criteria: were age ≥40 years, had a diagnostic code for COPD, had 2 years of continuous practice data (1-year baseline and 1-year outcome data), had at least 1 prescription for the salbutamol comparator or reference product during the outcome period (including the date when index prescriptions were received), and had not been prescribed the salbutamol comparator during the baseline period (S2 and S3 Figs).

Patients who received a prescription for the salbutamol reference during the baseline period and patients who did not receive any prescriptions for SABA nebulizers during the baseline period were also eligible for inclusion. In order to maximize the number of patients who regularly use the two drugs, patients with comorbid asthma were also included. Exclusion criteria included evidence of chronic respiratory diseases other than COPD/asthma and had ≥1 prescription for a SABA nebulizer other than the salbutamol reference within the baseline period. Patients could have used other maintenance therapies, including short-acting muscarinic antagonists (SAMAs), long-acting β_2_-agonists (LABAs), long-acting muscarinic antagonists (LAMAs), leukotriene receptor antagonists (LTRAs), inhaled corticosteroids (ICS), and theophylline (THEO), alone or in combination.

### Study outcomes

Our primary outcome was the rate of moderate and severe COPD exacerbations during the outcome period. Moderate COPD exacerbations were defined as an event in which the patient had a lower respiratory primary care consultation and received either an acute course of oral corticosteroids or an antibiotic prescription. Severe COPD exacerbations (hospitalizations) were defined as any event that resulted in a coded COPD-related emergency department (ED) or hospital admission or hospital admission on the same day as a lower respiratory consultation (excluding consult coding for a lung function test only).

Lower respiratory consultations were identified by lower respiratory diagnostic codes (including asthma, COPD, and lower respiratory tract infection diagnostic codes), asthma/COPD review codes (excluding any monitoring letter codes or lung function and/or asthma monitoring), and any additional respiratory examinations, referrals, chest x-rays, or outpatient consultations.

We examined 2 secondary outcomes. The rate of severe COPD exacerbations (hospitalizations; as defined above) during the outcome period was evaluated. The safety profiles of the salbutamol comparator and the salbutamol reference were analyzed by the rates of AEs defined in the Summary of Product Characteristics (SPC) for the salbutamol reference during the outcome period (ie, the 1-year period following the index prescription date) [9,15].

Adverse events were identified in the database by Read codes, classified by Medical Dictionary for Regulatory Activities (MedDRA) system organ class (SOC) (available at: http://www.meddra.org/) and compared for significant differences across matched cohorts for both the baseline and outcome periods. All AEs from the SPC list were evaluated and categorized by SOC in line with MedDRA standards. A report of an SPC-identified AE indicates that a consultation associated with the AE occurred.

### Statistical analyses

To validate the data and to establish whether the analysis would benefit from matching, we first conducted an exploratory analysis of baseline variables for the salbutamol comparator and reference cohorts (S1–S8 Tables). Evidence of patient comorbidities was calculated using the Charlson Comorbidity Index (CCI) score. The CCI is a method of predicting the 1-year mortality for a patient who may have a range of comorbid conditions where each condition is assigned a “weight” corresponding to the risk of death associated with the condition; scores are then summed to give a total score predicting mortality.

Based on differences identified through exploratory analysis of baseline variables, we matched individual patients from each cohort (1:1) to ensure comparison of similar patients. Exact matching for categorical variables and coarsened exact matching for numeric variables were used to match patients using 1:1 nearest neighbor matching, without replacement. Matching variables, such as demographic data, disease comorbidity, and indicators of disease severity were considered for selection using a combination of baseline data analysis and predictive modeling of baseline data in relation to the primary outcome variable (independently of treatment group). Pearson and Spearman correlation coefficients (as appropriate) were calculated between all baseline variables to determine strengths of linear relationships between variables. Correlation coefficients were considered to identify pairings of variables that may present collinearity issues at the modeling stage.

Multivariate analyses were carried out to identify baseline variables predictive (*P*<0.05) of outcomes. We considered these variables to be potential confounders when modeling the outcome variables. This robust statistical approach helped to minimize the potential confounding of results by indication or severity. Final matching criteria were age at receipt of index prescription, subcohort (patients who changed, continued, or initiated treatment at index prescription date), gender, baseline number of moderate and severe exacerbations, baseline SABA prescribed daily dosage, and baseline ICS, LAMA, and LABA use (Table 1).

**Table 1:** Matching criteria.

Criteria	Categories
Subcohort	Change/Continuing; Initiate/Initiate
Gender	Male/female
Baseline moderate and severe COPD exacerbations	0; 1; 2; 3+
Baseline SABA prescribed daily dosage (μg)	0; 1–200; 201–400; 401–600; 601–1000, and 1000+
ICS use in baseline	Yes/no
Baseline LAMA use	Yes/no
Baseline LABA use	Yes/no
Age at index prescription date	±5 years

COPD = chronic obstructive pulmonary disease; ICS = inhaled corticosteroid; LABA = long-acting β_2_-agonist; LAMA = long-acting muscarinic antagonist; SABA = short-acting β_2_-agonist.

Our previous research has demonstrated that 40.8% of COPD patients on SABA inhalers are expected to have an exacerbation within a 1-year period following treatment initiation [16]. To achieve adequate statistical power with a one-sided 0.05 significance level, sample sizes of 1122 patients per group were required. Previous randomized clinical studies evaluating efficacy and safety in COPD patients have reported that a 20% difference between treatment groups is clinically significant [17,18]. We chose a more stringent non-inferiority limit of <15% for the upper limit of the 95% confidence interval (CI) for the mean difference between treatments in all primary and secondary outcomes. Taken together, this enabled 90% power to show that there was no statistical difference between groups when the 95% CI upper limit of mean difference between treatments was <15%.

The primary outcome of non-inferiority for salbutamol comparator vs reference was established if the 95% CI upper limit of mean difference in proportions between treatments in the rate of moderate and severe exacerbations was <15%. Conditional Poisson regression models used empirical standard errors (for more conservative confidence interval estimations) and adjustments were made for potential baseline confounders. Rate ratios (RR) with 95% CI were used to compare salbutamol comparator and reference cohorts in rate of moderate and severe exacerbations and AEs. Summary statistics were carried out for all outcome variables (for matched patients) and consisted of sample size (number and percentage of non-missing values) and count and percentage by category (distribution).

No significant difference was observed between the treatments when the 95% CI included 1.

All study analyses were intent-to-treat based on ≥1 prescription for either salbutamol comparator or reference that determined the patient cohort placement. We conducted all statistical analyses using SPSS Statistics version 21 software (IBM SPSS Statistics, Feltham, Middlesex, United Kingdom) and SAS version 9.3 software (SAS Institute, Marlow, Buckinghamshire, United Kingdom).

## Results

### Demographic and clinical characteristics of matched cohorts

**Fig 1:**
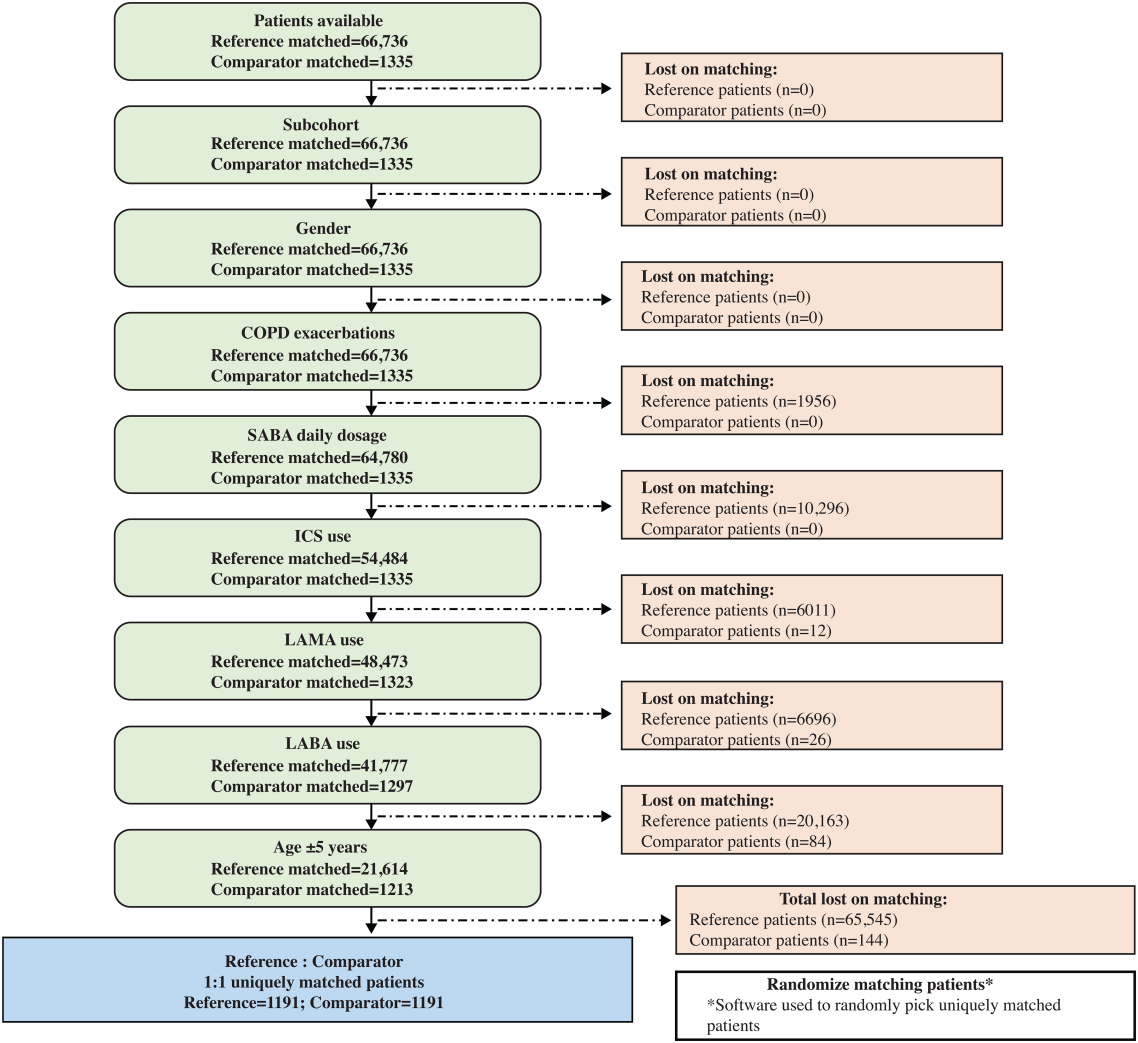
Patient matching flow chart. COPD = chronic obstructive pulmonary disease; ICS = inhaled corticosteroid; LABA = long-acting β_2_-agonist; LAMA = long-acting muscarinic antagonist; SABA = short-acting β_2_-agonist.

Following matching, 1191 patients were eligible for each of the 2 study cohorts (Fig 1). The median age of patients when they received the index prescription for the salbutamol comparator or reference was 68 years for both patient cohorts (range: 60–74 years). Approximately 46% of patients in each cohort were male (Table 2).

**Table 2:** Demographics and clinical characteristics of matched patients at baseline.

	Salbutamol Comparator	Salbutamol Reference	*P*-value (Conditional Logistic Regression)
	n = 1191	n = 1191	
Year index prescription was received, median (IQR)	2001 (1998, 2005)	1998 (1995, 2004)	<0.001
Age at index prescription date, median (IQR)	68 (60, 74)	68 (60, 74)	0.883
Age categories, n (%)			
40–50 y	83 (7.0)	86 (7.2)	0.990
>50–60 y	222 (18.6)	222 (18.6)	
>60–70 y	400 (33.6)	406 (34.1)	
>70–80 y	389 (32.7)	386 (32.4)	
>80 y	97 (8.1)	91 (7.6)	
Gender, n (%) Males	547 (45.9)	547 (45.9)	N/A
Smoking status, n (%)			
Non-smoker	215 (18.1)	198 (16.6)	
Current smoker	396 (33.2)	416 (34.9)	0.013
Ex-smoker	514 (43.2)	538 (45.2)	
Asthma diagnosis, n (%)	127 (10.7)	180 (15.1)	<0.001
Rhinitis diagnosis, n (%)	154 (12.9)	189 (15.9)	0.043
GERD diagnosis, n (%)	199 (16.7)	215 (18.1)	0.377
Ischemic heart disease diagnosis, n (%)	213 (17.9)	186 (15.6)	0.126
Diabetes diagnosis, n (%)	142 (11.9)	108 (9.1)	0.023
Prescribed NSAIDs, n (%)	447 (37.5)	423 (35.5)	0.294
Prescribed beta blockers, n (%)	58 (4.9)	47 (3.9)	0.270
Charlson Comorbidity Index score, n (%)			
0	511 (42.9)	411 (34.5)	<0.001
1–4	319 (26.8)	372 (31.2)	
≥5	361 (30.3)	408 (34.3)	
Moderate and severe COPD exacerbations in the year before and including index prescription date was received, n (%)			
0	486 (40.8)	486 (40.8)	N/A
1	278 (23.3)	278 (23.3)	
2	178 (14.9)	178 (14.9)	
≥3	249 (20.9)	249 (20.9)	
Severe COPD exacerbations in the year before and including index prescription date, n (%)			
0	1119 (94.0)	1120 (94.0)	1
≥1	71 (6.0)	71 (6.0)	
FEV_1_% predicted,^a^ median (IQR)	48.73 (35.60, 63.02)	46.17 (33.37, 63.00)	0.076
Distribution of patients among categories of FEV_1_% predicted, n (%)^b^			
<30 (very severe)	109 (15.4)	120 (18.8)	0.027
30–49 (severe)	262 (37.0)	238 (37.2)	
50–79 (moderate)	261 (36.9)	226 (35.3)	
≥80 (mild)	76 (10.7)	56 (8.8)	
Any AE, n (%)			
0	935 (78.5)	943 (79.2)	0.862
1	162 (13.6)	151 (12.7)	
≥2	94 (7.9)	97 (8.1)	

AE = adverse event; COPD = chronic obstructive pulmonary disease; FEV_1_ = forced expiratory volume in 1 second; GERD = gastroesophageal reflux disease; IQR = interquartile range; NSAIDs = non-steroidal anti-inflammatory drugs; y = year.

^a^Recorded closest to date when index prescription was received.

^b^Percent of non-missing patients.

Data for unmatched cohorts are provided in S1, S2, S3 and S7 Tables.

Despite patient matching, some small differences in baseline characteristics remained between groups (Table 2). Significant between-group differences included the year in which the index prescription for salbutamol comparator or reference was received, smoking status, the prevalence of asthma, rhinitis, or diabetes as comorbidities, Charlson Comorbidity Index scores, and the distribution of patients among categories of forced expiratory volume in 1 second (FEV_1_). Outcome analyses were performed after adjustment for these residual differences. No significant differences were present between cohorts in baseline use of respiratory medications (Table 3) or in baseline AEs (S9 Table). However, a significant difference in drug strength at the date of the index prescription was detected, with 54.2% and 43.0% of the salbutamol comparator and reference cohorts receiving the 2.5 mg/2.5 mL dose, respectively. The remaining patients, including 45.8% of patients in the salbutamol comparator group and 57.0% of patients in the reference group, received the 5.0 mg/2.5 mL dose (*P*<0.001).

**Table 3:** Use of respiratory therapies in matched patients at baseline.

	Salbutamol Comparator n = 1191	Salbutamol Reference n = 1191	*P*-value (Conditional Logistic Regression)
≥1 prescription for respiratory therapies in the year before date when index prescription was received, n (%)			
SABA Nebulizers	138 (11.6)	138 (11.6)	N/A
ICS	916 (76.9)	916 (76.9)	N/A
LABA	296 (24.9)	296 (24.9)	N/A
LAMA	137 (11.5)	137 (11.5)	N/A
LTRA	49 (4.1)	38 (3.2)	0.229
SAMA	396 (33.2)	391 (32.8)	0.819
THEO	286 (24.0)	253 (21.2)	0.092
Prescriptions for acute oral corticosteroids* for lower respiratory event in year before date when index prescription was received, n (%)			
0	710 (59.6)	699 (58.7)	0.411
1	242 (20.3)	239 (20.1)	
≥2	239 (20.1)	253 (21.3)	
Prescriptions for antibiotics for lower respiratory event in the year before date when index prescription was received, n (%)			
0	592 (49.7)	596 (50.0)	0.445
1	260 (21.8)	255 (21.4)	
≥2	339 (28.5)	340 (18.5)	

ICS = inhaled corticosteroid; LABA = long-acting β_2_-agonist; LAMA = long-acting muscarinic antagonist; LTRA = leukotriene receptor antagonist; SABA = short-acting β_2_-agonist; SAMA = short-acting muscarinic antagonist; THEO = theophylline.

*All courses of oral corticosteroids that are definitely not maintenance therapy and/or all courses where dosing instructions suggest exacerbation treatment (e.g. 6000–1000 reducing, or 30000 μg as directed) and/or all courses with no dosing instructions, but unlikely to be maintenance therapy, with a code for COPD or a lower respiratory event.

Data for unmatched cohorts are provided in S4, S5 and S6 Tables.

### Clinical outcomes

**Fig 2:**
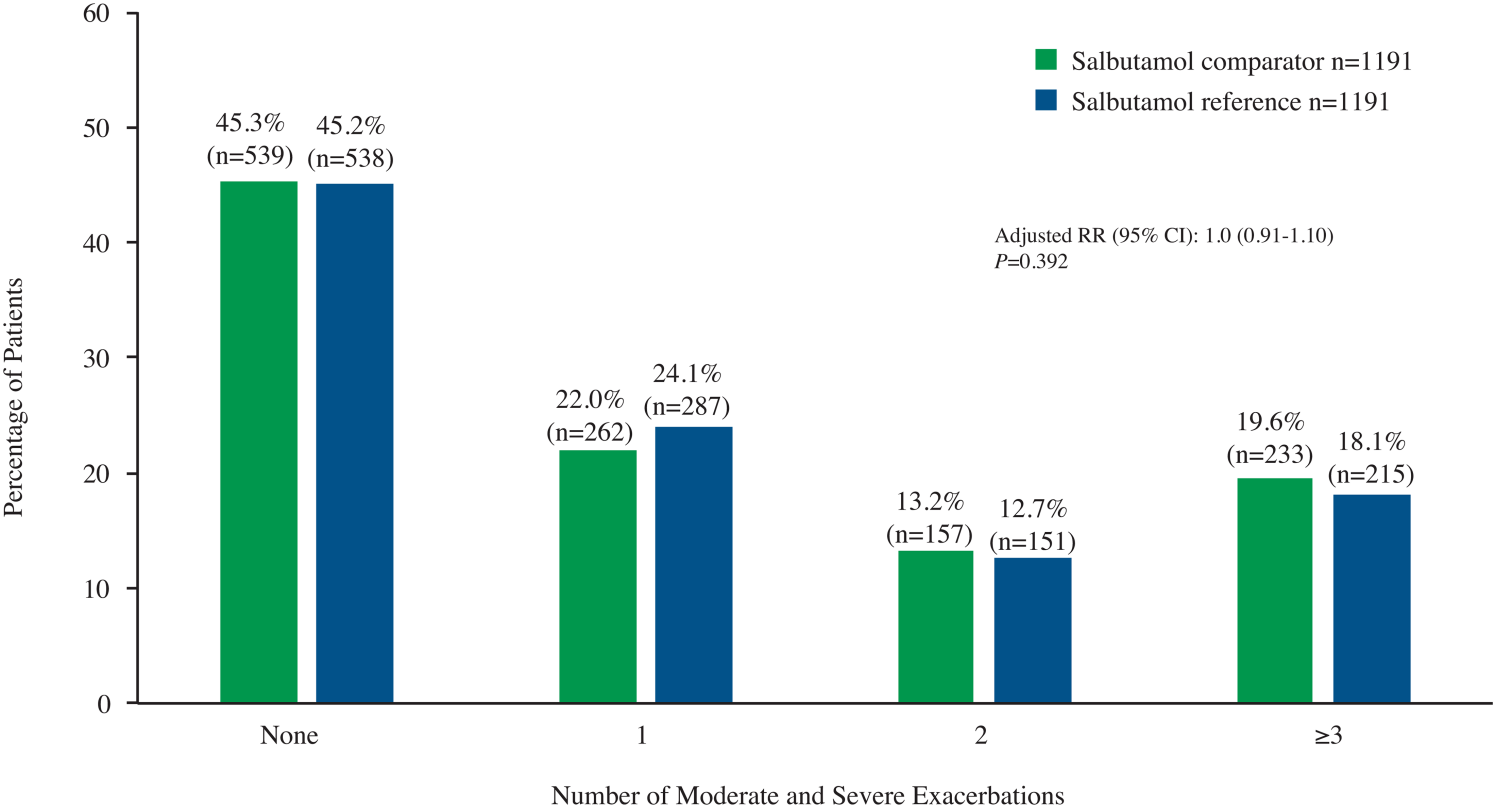
Incidence of moderate and severe exacerbations. RR = rate ratio.

The salbutamol comparator was non-inferior to the salbutamol reference for the primary outcome of rate of moderate and severe exacerbations over the outcome period, with an adjusted upper 95% CI for the difference in proportions for the comparator vs the reference of 0.032 (3.2%), which was below the non-inferiority criterion of <15%. Similarly, no significant difference between treatment groups was observed in the subcohort of patients who initiated treatment with either agent at baseline. In this subanalysis, the adjusted upper 95% CI for the difference in proportions for the salbutamol comparator vs the reference was 3.7%. Moreover, no significant difference between treatment groups was observed for the rate of moderate and severe exacerbations in the overall population. The adjusted RR for the rate of moderate and severe exacerbations for the salbutamol comparator versus the reference was 1.0 (95% CI: 0.91, 1.10) (Fig 2).

**Fig 3:**
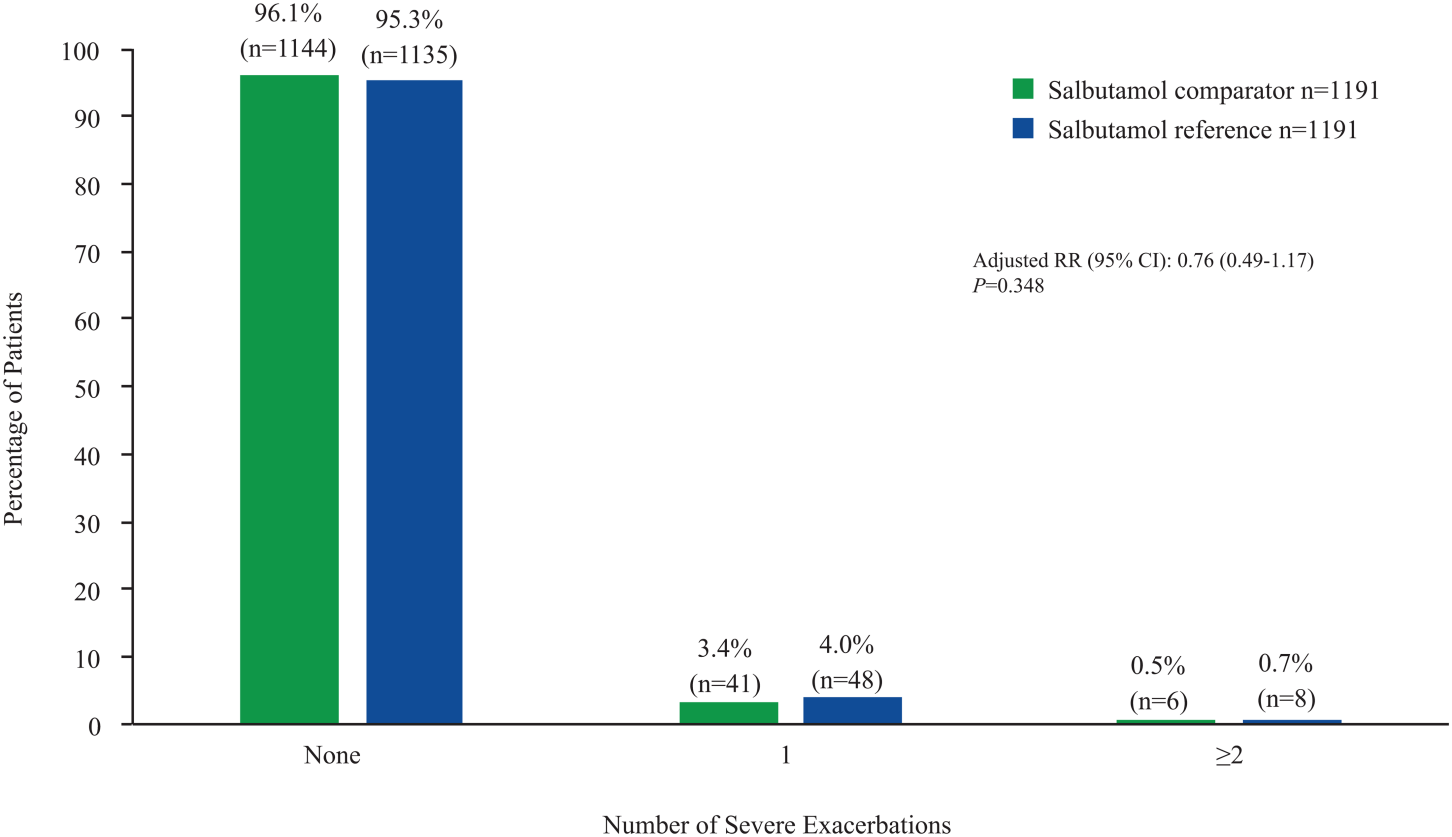
Incidence of severe exacerbations. RR = rate ratio.

No significant difference was found in the rate of severe exacerbations during the outcome period following prescriptions with the salbutamol comparator vs the reference (RR: 0.76 [95% CI: 0.49, 1.17]) (Fig 3). No significant difference was observed between the treatments in the rate of severe exacerbations over the outcome period in both the overall population and the initiation subcohort.

**Fig 4:**
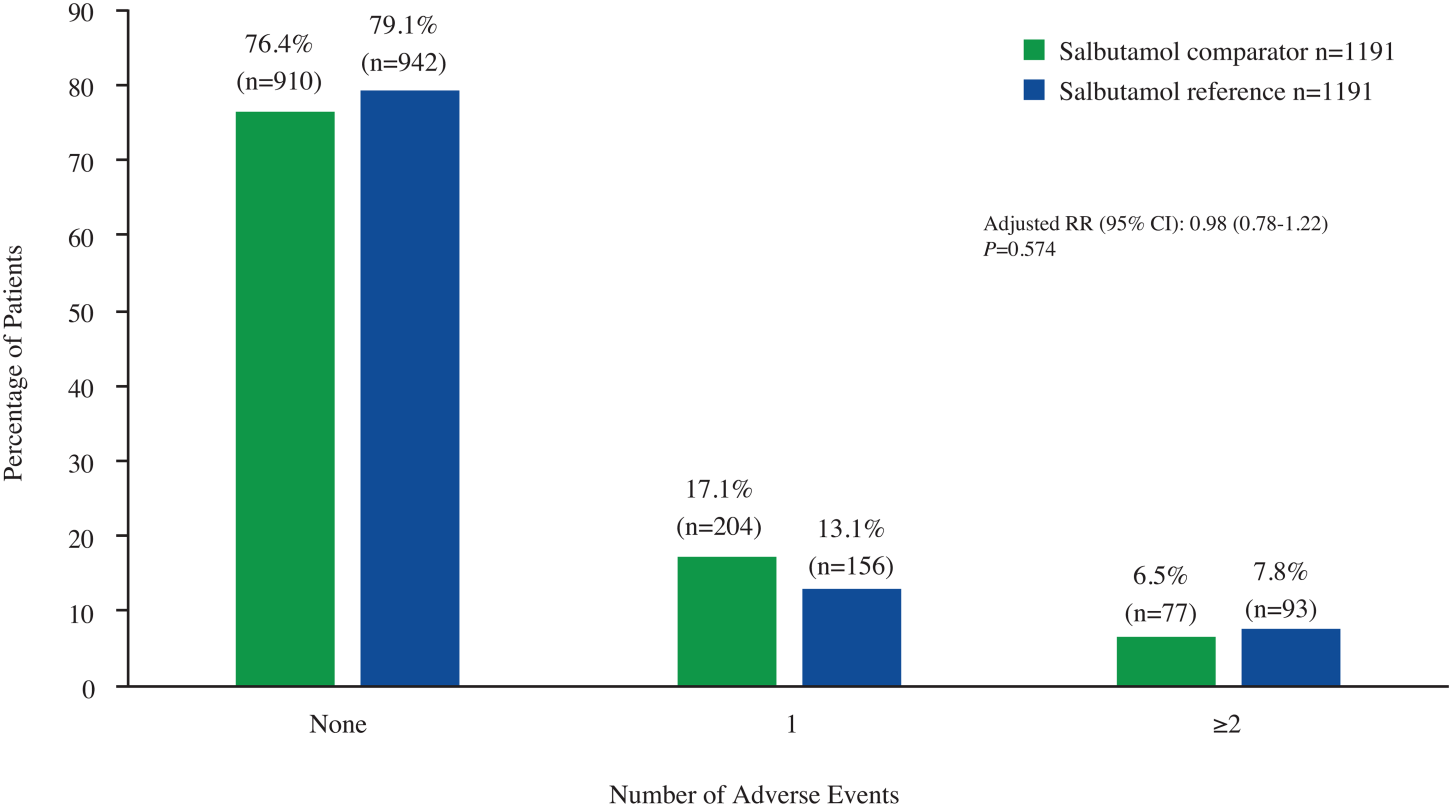
Incidence of any adverse event. RR = rate ratio.

Results for any AE (yes/no) indicated no significant difference between treatment groups over the 1-year outcome period (Fig 4). Additionally, no significant differences between groups were detected for the incidence of AEs by SOC (Table 4) or for any individual AE (Table 5). A total of 13 patients died during the 1-year outcome period: 7 patients (0.6%) from the salbutamol comparator treatment group and 6 patients (0.5%) from the reference group (*P*<0.782).

**Table 4:** Individual AE categories during the outcome year in matched patients.

	Salbutamol Comparator n = 1191	Salbutamol Reference n = 1191	*P*-value (Conditional Poisson Regression)
Immune system disorders (angioedema, urticaria, bronchospasm, hypotension, collapse), n (%)			
None	1153 (96.8)	1163 (97.6)	0.847
1	33 (2.8)	15 (1.3)	
≥2	5 (0.4)	13 (1.1)	
RR (95% CI)	0.69 (0.40, 1.18)	1.00	
Metabolic and nutritional disorders (hypokalemia), n (%)			
None	1189 (99.8)	1187 (99.7)	0.340
1	2 (0.2)	3 (0.3)	
≥2	0 (0.0)	1 (0.1)	
RR (95% CI)^a^	0.40 (0.71, 2.26)	1.00	
Nervous system disorders (tremor, headache), n (%)			
None	1157 (97.1)	1152 (96.7)	0.342
1	30 (2.5)	30 (2.5)	
≥2	4 (0.3)	9 (0.8)	
RR (95% CI)	0.65 (0.39, 1.09)	1.00	
Cardiovascular disorders (atrial fibrillation, tachycardia, extrasystoles, IHD, palpitations), n (%)			
None	1031 (86.6)	1050 (88.2)	0.476
1	116 (9.7)	94 (7.9)	
≥2	44 (3.7)	47 (3.9)	
RR (95% CI)	1.08 (0.78, 1.50)	1.00	
Respiratory, thoracic, and mediastinal disorders (bronchospasm/paradoxical bronchospasm), n (%)			
None	1170 (98.2)	1175 (98.7)	1.00
1	18 (1.5)	8 (0.7)	
≥2	3 (0.3)	8 (0.7)	
RR (95% CI)	0.73 (0.36, 1.48)	1.00	
Gastrointestinal disorders (mouth and throat irritation), n (%)			
None	1135 (95.3)	1138 (95.5)	1.00
1	47 (3.9)	41 (3.4)	
≥2	9 (0.8)	12 (1.0)	
RR (95% CI)	1.10 (0.74, 1.64)	1.00	
Musculoskeletal and connective tissue disorders (muscle cramps), n (%)			
None	1172 (98.4)	1171 (98.3)	1.00
1	17 (1.4)	19 (1.6)	
≥2	2 (0.2)	1 (0.1)	
RR (95% CI)	0.95 (0.50, 1.80)	1.00	

AE = adverse event; CI = confidence interval; IHD = ischemic heart disease; RR = rate ratio.

^a^Unadjusted for baseline confounders.

Data for unmatched cohorts are provided in S8 Table.

**Table 5:** Individual AEs recorded in the outcome period in the main analysis, n (%).

	Salbutamol Comparator (n = 1191)	Salbutamol Reference (n = 1191)	*P*-value (Conditional Poisson Regression)
Ischemic heart disease	125 (10.5)	107 (9)	0.213
Mouth and throat irritation	56 (4.7)	53 (4.5)	0.770
Cardiac arrhythmias*	45 (3.8)	39 (3.3)	0.502
Headache	28 (2.4)	32 (2.7)	0.382
Bronchospasm/paradoxical bronchspasm	21 (1.8)	16 (1.3)	0.400
Muscle cramps	19 (1.6)	20 (1.7)	0.873
Tremor	6 (0.5)	8 (0.7)	0.597
Urticaria	5 (0.4)	6 (0.5)	0.763
Angioedema	3 (0.3)	2 (0.2)	0.654
Peripheral vasodilation	2 (0.2)	0 (0)	-
Hypokalemia	2 (0.2)	4 (0.3)	0.423
Collapse	9 (0.8)	4 (0.3)	0.177

AE = adverse event.

*Including atrial fibrillabion, tachycardia, extrasystoles, and palpitations.

Data for unmatched cohorts are provided in S8 and S9 Tables.

## Discussion

Effective treatment and prevention of COPD exacerbations is a central clinical practice objective. COPD exacerbation is associated with declines in lung and physical function, increased risk for hospitalization and further health decline, increased risk for subsequent exacerbations, and significant healthcare costs [7,10,11]. Patients with moderate COPD have shown prolonged impairments in quality of life, including social and emotional functioning, following exacerbations [19]. In addition to minimizing decline in lung function and preventing hospitalization, effective exacerbation treatment is also key to prevention of subsequent exacerbations [3,6].

The primary objective of our study was to determine whether the effectiveness of the salbutamol comparator was non-inferior to that of the reference product in a broad population of patients with COPD managed in real-life primary care practices in the UK. The data collected enabled us to compare the impact of these medications, together with concomitant respiratory medications during real-life clinical management of COPD, on rates of moderate and severe exacerbations and reported AEs in the absence of a randomized, head-to-head comparison. Our findings demonstrated that rates of moderate and severe exacerbations and hospitalizations resulting from COPD exacerbations with the salbutamol comparator were non-inferior to those reported with the salbutamol reference. No significant differences were observed for rates of moderate and severe exacerbations or for severe exacerbations between the salbutamol comparator and reference. No clinically significant differences were observed between treatments for any AE and for AEs by SOC.

By examining a large and diverse population of patients with COPD, including those with a wide range of comorbidities, ages, and COPD severities, we attempted to represent the broad spectrum of patients with COPD typically encountered in primary care practices. One of the strengths of our study is that we were able to include nearly 1200 patients in each treatment group and were able to examine the outcomes associated with these therapies in the context of real-life COPD management. We also reviewed a full 2 years of data– 12 months before and after the date of the index prescription. In contrast, most randomized controlled trials are often conducted over shorter durations, have strict selection criteria, resulting in a homogeneous patient population that may not be representative of real-life clinical practices [20,21]. We were also able to examine outcomes in patients who were naïve to treatment with either salbutamol formulation and those who had previously received the salbutamol reference and either continued with the reference or switched to the comparator, in patients using a variety of other respiratory therapies, giving us the opportunity to evaluate outcomes in most of the real-life scenarios in which these formulations would be used.

A drawback of our inclusion of a broad and diverse patient population is the small differences in baseline characteristics that inevitably remain between treatment groups, despite the fact that we matched patients using several clinically important variables. For example, more patients in the salbutamol reference group had ongoing active comorbid asthma and rhinitis, and Charlson Comorbidity Index scores were also generally higher among patients in the reference group, indicating that patients in the reference group had more comorbidities than patients in the salbutamol comparator group. These residual differences were managed statistically in the current study outcome analyses.

Patients with comorbid asthma were included in the study in order to maximize the number of patients who routinely use the two drugs. However, since salbutamol is prescribed for both COPD and asthma, the condition for which the prescription was written cannot be determined. In addition, inclusion of patients with both conditions may have also confounded accurate identification of COPD exacerbations versus asthma exacerbations.

In addition, patients in the reference group had slightly more diminished lung function than patients in the comparator group. It should be noted, though, that patients were matched for the baseline number of moderate and severe exacerbations, and thus disease severity, in terms of exacerbations, was similar between the groups at baseline. The slight difference in baseline lung function may explain the significant difference between groups in drug strength of the index prescription. Significantly more patients on the salbutamol reference versus the comparator received the higher dose (5.0 mg/2.5 mL) during the outcome period (57.0% vs 45.8%; *P*<0.001). While small differences in ICS use, LABA use, and LAMA use at baseline were noted before matching, no differences in these important variables were seen between groups after matching, suggesting that differences in the use of maintenance or controller therapies do not explain the differences in prescribed dosages of salbutamol between groups. Despite these differences, the salbutamol comparator cohort rates of moderate or severe exacerbations were non-inferior to those observed in the reference group. Additionally, the rate of severe exacerbations (hospitalizations) did not differ between the comparator and the reference group.

It is also important to note that the overall rates of AEs reported in the salbutamol comparator group were comparable to those reported in the reference group. Similarly, rates of AEs analyzed by SOC were also similar between groups. Moreover, no statistically or clinically significant between-group differences were observed in the rates of individual AEs (as documented in the Summary of Product Characteristics for the salbutamol comparator product), serious AEs, or deaths, indicating that both agents have comparable safety profiles. However, due to the complexity of the database, it should be noted that the SPC-listed AEs used to indicate AEs during treatment represent proxies for potential AEs rather than verified treatment-related AEs. Although an AE report indicates that a consultation associated with the SPC-identified AE occurred, the actual cause of the AE may or may not be related to treatment, the underlying disease, or even another pre-existing condition. As a result, AEs are likely to be over-reported.

As for any retrospective study, only data collected can be analyzed. Indicators such as changes in FEV_1_, disease progression, changes in other respiratory therapies, and cost were not collected or analyzed. Although the primary care practice databases we analyzed do not include prescriptions outside of general or primary care practices, the majority of prescriptions in the UK are provided within primary care. It should also be noted that while our data do include information on prescriptions for the salbutamol comparator and reference products, they do not provide information on actual drug use. It cannot be assumed that the drugs prescribed were actually used in a manner consistent with the prescription. Our real-life clinical management patient cohorts were determined by ≥1 prescription for either comparator or reference product and an intent-to-treat analysis approach was used. This was appropriate for our safety analyses but did not allow for examination of the number of prescriptions to estimate frequency of use or timing of use of comparator or reference products. Whereas SABAs have been shown to effectively treat exacerbations, and effective exacerbation treatment is known to prevent subsequent exacerbations [3,6], the direct role of SABAs in preventing exacerbations has not yet been demonstrated. In our study, the possibility that the comparator and reference products were used for treatment of exacerbations rather than prevention of exacerbations could not be evaluated. Examination of frequency and timing of prescriptions relative to exacerbations is warranted to address this question in future studies. Additional questions of interest for future studies include comparison of the efficacy of the comparator and reference drugs with SABA metered dose aerosol inhaler, and the reasons underlying physician choice of one prescription over another. We can hypothesize multiple potential factors in addition to availability that may drive physician selection of a given drug, including cost, what drug options are on the prescribing formulary, the AE profile of the drug, patient preference, and prior prescribing experience of the physician with the drug.

Given the inherent limitations of database studies, such as potential confounding factors with internal validity, results from this study should ideally be considered in conjunction with those of randomized controlled trials. Nevertheless, our results indicate that salbutamol delivered via nebulizer as either the salbutamol comparator or reference is safe and effective in patients with COPD. These findings are consistent with results from previous studies evaluating salbutamol in patients with COPD [22].

## Conclusion

There were no significant differences in moderate and severe COPD exacerbation and AE rates between the matched cohorts of salbutamol comparator and reference treatment groups. These results were confirmed by subanalysis of the 2 initiation subcohorts. It can therefore be concluded that the salbutamol comparator is non-inferior to the salbutamol reference and is an effective treatment alternative, together with concomitant respiratory medications typical of real-life clinical management of COPD. Its safety profile is comparable to that of the reference product.

Owing to the rising costs of COPD management [10–12,23], the availability of the lower-cost generic treatment salbutamol comparator may provide an affordable, safe, and useful alternative to the salbutamol reference in patients with COPD managed in primary care practices.

## Supporting information

S1 Table Demographics in the unmatched patient population. BMI = body mass index; IQR = interquartile range. *Patients may be included more than once with a different index prescription date. Number of unique patients is 7938. ^†^Mann-Whitney. ^‡^BMI categories: Underweight: <18.5; Normal: 18.5–24.9; Overweight: 25.0–29.9; Obese: ≥30.0.
(DOCX)

S2 Table Comorbidities and comedications in the unmatched patient population. CCI = Charlson Comorbidity Index; GERD = gastroesophageal reflux disease; IHD = ischemic heart disease; NSAIDs = nonsteroidal anti-inflammatory drugs. *Patients may be included more than once with a different index prescription date. Number of unique patients is 7938. ^†^Quality and Outcomes Framework (QOF) Read codes (excluding patients with asthma resolved codes) recorded in the year after the index prescription date. ^‡^Prescriptions received during the 1 year prior to (and including) the index prescription date. ^§^Calculated using the Charlson Comorbidity Index (ICD-9 codes translated to ICD-10 for UK use) over the 1 year prior to (and including) the index prescription date.
(DOCX)

S3 Table Lung function in the unmatched patient population. FEV_1_ = forced expiratory volume in 1 second; GOLD = Global Initiative for Chronic Obstructive Lung Disease; IQR = interquartile range. *Patients may be included more than once with a different index prescription date. Number of unique patients is 7938. ^†^Mann-Whitney. ^‡^Very severe: FEV_1_ is <30% of predicted value; severe: FEV_1_ is between 30%-49% of predicted value; moderate: FEV_1_ is between 50%-79% of predicted value; mild FEV_1_ is ≥80% of predicted value. ^§^Based on Global Initiative for Chronic Obstructive Lung Disease (GOLD) Guidelines 2011: A = Low risk, low symptom burden (mMRC of 0–1) AND FEV_1_ of 50% or greater (old GOLD 1–2) AND/OR low exacerbation rate (0-1/year); B = Low risk, higher symptom burden (mMRC of 2 or more) AND FEV_1_ of 50% or greater (old GOLD 1–2) AND/OR low exacerbation rate (0-1/year); C = High risk, low symptom burden (mMRCof 0–1) AND FEV_1_ <50% (old GOLD 3–4) AND/OR high exacerbation rate (2 or more/year); D = High risk, higher symptom burden (mMRC of 2 or more) AND FEV_1_ <50% (old GOLD 3–4) AND/OR high exacerbation rate (2 or more/year). Both routine medical practice recorded and patient questionnaire mMRC scores were used, with the most recent score taking precedence.
(DOCX)

S4 Table Use of SABAs at baseline in the unmatched patient population. IQR = interquartile range; SABA = short-acting β_2_-agonist. *Patients may be included more than once with a different index prescription date. Number of unique patients is 7938. ^†^Mann-Whitney. ^‡^Daily dose calculated as: (count of inhalers * doses in pack) / 365) * μg strength.
(DOCX)

S5 Table Use of other respiratory medications at baseline in the unmatched patient population. ICS = inhaled corticosteroid; LABA = long-acting beta agonist; LAMA = long-acting muscarinic anatagonist; LTRA = leukotriene antagonist; SABA = short-acting β_2_-agonist; SAMA = short-acting muscarinic antagonist; THEO = theophylline. *Patients may be included more than once with a different index prescription date. Number of unique patients is 7938. ^†^Salbutamol reference. Applies to changing (within the comparator cohort) and continuing (within the reference cohort) subcohorts only. ^‡^All courses that are definitely not maintenance therapy and/or all courses where dosing instructions suggest exacerbation treatment (eg, 6–1 reducing, or 30 mg as directed) and/or all courses with no dosing instructions, but unlikely to be maintenance therapy, with a code for COPD or a lower respiratory event. ^§^Lower respiratory diagnostic codes (including asthma, COPD and lower respiratory tract infection (LRTI) read codes) or asthma/COPD review codes excluding any monitoring letter codes or lung function and/or asthma monitoring AND any additional respiratory examinations, referrals, chest x-rays, or events.
(DOCX)

S6 Table Use of maintenance therapies at baseline in the unmatched patient population. ICS = inhaled corticosteroid; LABA = long-acting beta agonist; LAMA = long-acting muscarinic anatagonist; LTRA = leukotriene antagonist; SABA = short-acting β_2_-agonist; SAMA = short-acting muscarinic antagonist; THEO = theophylline. *Patients may be included more than once with a different index prescription date. Number of unique patients is 7938.
(DOCX)

S7 Table Exacerbation history at baseline in the unmatched patient population. COPD = chronic obstructive pulmonary disease. *Patients may be included more than once with a different index prescription date. Number of unique patients is 7938.
(DOCX)

S8 Table AEs at baseline in the unmatched patient population. AE = adverse event; IHD = ischemic heart disease. *Patients may be included more than once with a different index prescription date. Number of unique patients is 7938. ^†^Includes atrial fibrillation, tachycardia, extrasystoles, and palpitations. ^‡^Due to small numbers chi-square results may be invalid.
(DOCX)

S9 Table AE reports in matched cohorts at baseline. AE = adverse event; CLR = conditional logistical regression; IHD = ischemic heart disease. Data are expressed as number (%) of patients who had no AEs recorded or had them recorded at least once in the year prior to the index prescription date. *Includes atrial fibrillation, tachycardia, extrasystoles, and palpitations.
(DOCX)

S1 Fig Study design. SABA = short-acting β_2_-agonist; UK = United Kingdom. *A 1-year period enabled the recording of any measurable change in outcomes and allowed for seasonal changes in respiratory diseases and their related conditions. ^†^Based on evaluation of the differences between cohorts during the baseline period, patients from the 2 cohorts were matched on demographics and clinical characteristics to ensure comparison of similar patients. Patients initiating the reference were matched to patients initiating the comparator and patients in the change subcohort were matched to patients in the continuing subcohort. ^‡^Patients could be included more than once with different index prescription dates if they satisfied the inclusion/exclusion criteria. However, during the matching process, it was ensured that only unique patients were analyzed.
(EPS)

S2 Fig Patient population flow chart for patients prescribed the salbutamol reference product. COPD = chronic obstructive pulmonary disease; CPRD = Clinical Practice Research Datalink; IPD = index prescription date; OPCRD = Optimum Patient Care Research Database; SABA = short-acting β_2_-agonist.
(EPS)

S3 Fig Patient population flow chart for patients prescribed the comparator product. COPD = chronic obstructive pulmonary disease; CPRD = Clinical Practice Research Datalink; IPD = index prescription date; OPCRD = Optimum Patient Care Research Database; SABA = short-acting β_2_-agonist.
(EPS)
