# Supplementary material for: Real-life effectiveness and safety of salbutamol Steri-Neb™ vs. Ventolin Nebules® for exacerbations in patients with COPD: Historical cohort study
Source: PLoS One. 2018 Jan 24;13(1):e0191404. doi: 10.1371/journal.pone.0191404 (PMC5783390; doi:10.1371/journal.pone.0191404)
Supplement: S1 Table — BMI = body mass index; IQR = interquartile range. *Patients may be included more than once with a different index prescription date. Number of unique patients is 7938. †Mann-Whitney. ‡BMI categories: Underweight: <18.5; Normal: 18.5–24.9; Overweight: 25.0–29.9; Obese: ≥30.0. (DOCX) [file pone.0191404.s001.docx]

|  |  | **Unmatched cohorts** | | |
| --- | --- | --- | --- | --- |
|  |  | **Salbutamol Comparator**  **(n=1335)** | **Salbutamol  Reference**  **(n=66,736)**^*^ | ***P*-value**  **(Chi-square)** |
| Year of index prescription | Median (IQR) | 2001 (1998; 2005) | 1999 (1995; 2003) | <0.001^†^ |
| Age at index prescription | Median (IQR) | 68 (60;75) | 69 (61;75) | 0.031^†^ |
| Distribution of patients among age categories, n (%) | 40-50 | 96 (7.2) | 4682 (7) | 0.422 |
|  | >50-60 | 253 (19) | 11,365 (17) |  |
|  | >60-70 | 438 (32.8) | 22,120 (33.1) |  |
|  | >70-80 | 421 (31.5) | 21,966 (32.9) |  |
|  | >80 | 127 (9.5) | 6603 (9.9) |  |
| Gender, n (%) | Males | 622 (46.6) | 31,221 (46.8) | 0.89 |
| Smoking status (recorded closest to index prescription date), n (%) | Non-missing | 1292 (96.8) | 62,265 (93.3) | _ |
|  | Non-smoker | 223 (16.7) | 12,081 (18.1) | <0.001 |
|  | Current smoker | 456 (34.2) | 17,696 (26.5) |  |
|  | Ex-smoker | 613 (45.9) | 32,517 (48.7) |  |
| BMI (kg/m^2^) category (closest to index prescription date), n (%)^‡^ | Non-missing | 1143 (85.6) | 57,193 (85.7) | - |
|  | Median (IQR) | 26.17 (23.03; 30.46) | 25.80 (22.58; 29.75) | <0.001^†^ |
|  | Underweight | 54 (4.4) | 2752 (4.8) | 0.026 |
|  | Normal weight | 441 (36.0) | 22,302 (39.0) |  |
|  | Overweight | 396 (32.4) | 18,507 (32.4) |  |
|  | Obese | 333 (27.2) | 13,559 (23.7) |  |
