# Supplementary material for: Real-life effectiveness and safety of salbutamol Steri-Neb™ vs. Ventolin Nebules® for exacerbations in patients with COPD: Historical cohort study
Source: PLoS One. 2018 Jan 24;13(1):e0191404. doi: 10.1371/journal.pone.0191404 (PMC5783390; doi:10.1371/journal.pone.0191404)
Supplement: S2 Table — CCI = Charlson Comorbidity Index; GERD = gastroesophageal reflux disease; IHD = ischemic heart disease; NSAIDs = nonsteroidal anti-inflammatory drugs. *Patients may be included more than once with a different index prescription date. Number of unique patients is 7938. †Quality and Outcomes Framework (QOF) Read codes (excluding patients with asthma resolved codes) recorded in the year after the index prescription date. ‡Prescriptions received during the 1 year prior to (and including) the index prescription date. §Calculated using the Charlson Comorbidity Index (ICD-9 codes translated to ICD-10 for UK use) over the 1 year prior to (and including) the index prescription date. (DOCX) [file pone.0191404.s002.docx]

|  |  | **Unmatched cohorts** | | |
| --- | --- | --- | --- | --- |
|  |  | **Salbutamol Comparator**  **(n=1335)** | **Salbutamol Reference**  **(n=66,736)*** | ***P*-value**  **(Chi-square)** |
| Patients with ongoing active comorbid asthma, n (%)† | No | 1130 (84.6) | 57,494 (86.2) | 0.115 |
|  | Yes | 205 (15.4) | 9242 (13.8) |  |
| Patients with rhinitis diagnosis, n (%) | No | 1156 (86.6) | 55,191 (82.7) | <0.001 |
|  | Yes | 179 (13.4) | 11,545 (17.3) |  |
| Patients with GERD diagnosis, n (%) | No | 1107 (82.9) | 55,345 (82.9) | 0.992 |
|  | Yes | 228 (17.1) | 11,391 (17.1) |  |
| Patients with IHD diagnosis, n (%) | No | 1082 (81) | 56,926 (85.3) | <0.001 |
|  | Yes | 253 (19.0) | 9810 (14.7) |  |
| Patients with diabetes diagnosis, n (%) | No | 1177 (88.2) | 60,053 (90.0) | 0.028 |
|  | Yes | 158 (11.8) | 6683 (10.0) |  |
| Patients prescribed NSAIDs, n (%)^‡^ | No | 825 (61.8) | 44,441 (66.6) | <0.001 |
|  | Yes | 510 (38.2) | 22,295 (33.4) |  |
| Patients prescribed beta-blockers,  n (%)^‡^ | No | 1266 (94.8) | 65,747 (98.5) | <0.001 |
|  | Yes | 69 (5.2) | 989 (1.5) |  |
| Distribution of patients among CCI score,^§^ categories, n (%) | 0 | 563 (42.2) | 14,810 (22.2) | <0.001 |
|  | 1-4 | 361 (27.0) | 24,175 (36.2) |  |
|  | 5+ | 411 (30.8) | 27,751 (41.6) |  |
