# Supplementary material for: Real-life effectiveness and safety of salbutamol Steri-Neb™ vs. Ventolin Nebules® for exacerbations in patients with COPD: Historical cohort study
Source: PLoS One. 2018 Jan 24;13(1):e0191404. doi: 10.1371/journal.pone.0191404 (PMC5783390; doi:10.1371/journal.pone.0191404)
Supplement: S3 Table — FEV1 = forced expiratory volume in 1 second; GOLD = Global Initiative for Chronic Obstructive Lung Disease; IQR = interquartile range. *Patients may be included more than once with a different index prescription date. Number of unique patients is 7938. †Mann-Whitney. ‡Very severe: FEV1 is <30% of predicted value; severe: FEV1 is between 30%-49% of predicted value; moderate: FEV1 is between 50%-79% of predicted value; mild FEV1 is ≥80% of predicted value. §Based on Global Initiative for Chronic Obstructive Lung Disease (GOLD) Guidelines 2011: A = Low risk, low symptom burden (mMRC of 0–1) AND FEV1 of 50% or greater (old GOLD 1–2) AND/OR low exacerbation rate (0-1/year); B = Low risk, higher symptom burden (mMRC of 2 or more) AND FEV1 of 50% or greater (old GOLD 1–2) AND/OR low exacerbation rate (0-1/year); C = High risk, low symptom burden (mMRCof 0–1) AND FEV1 <50% (old GOLD 3–4) AND/OR high exacerbation rate (2 or more/year); D = High risk, higher symptom burden (mMRC of 2 or more) AND FEV1 <50% (old GOLD 3–4) AND/OR high exacerbation rate (2 or more/year). Both routine medical practice recorded and patient questionnaire mMRC scores were used, with the most recent score taking precedence. (DOCX) [file pone.0191404.s003.docx]

|  | | **Unmatched cohorts** | | |
| --- | --- | --- | --- | --- |
|  | | **Salbutamol Comparator (n=1335)** | **Salbutamol Reference (n=66,736)*** | ***P*-value (Chi-square)** |
| FEV_1_ % predicted (recorded closest to index prescription date) | Non-missing | 809 (60.6) | 35,570 (53.3) | - |
|  | Median (IQR) | 48.64 (35.14; 63.03) | 41.49 (30.6; 57.38) | <0.001† |
| Distribution of patients among categories of FEV_1_ % predicted‡,  n (% of non-missing) | <30 (very severe) | 127 (15.7) | 7929 (23.8) | <0.001 |
|  | 30-49 (severe) | 299 (37.0) | 13,490 (40.5) |  |
|  | 50-79 (moderate) | 298 (36.8) | 9359 (28.1) |  |
|  | ≥80 (mild) | 85 (10.5) | 2523 (7.6) |  |
| GOLD group§,  n (% of non-missing) (recorded closest to index prescription date) | Non-missing | 778 (53.0) | 27,629 (41.4) | - |
|  | A | 226 (32.0) | 9219 (33.4) | 0.242 |
|  | B | 98 (13.9) | 4065 (14.7) |  |
|  | C | 229 (32.4) | 7976 (28.9) |  |
|  | D | 154 (21.8) | 6369 (23.1) |  |
