# Supplementary material for: Real-life effectiveness and safety of salbutamol Steri-Neb™ vs. Ventolin Nebules® for exacerbations in patients with COPD: Historical cohort study
Source: PLoS One. 2018 Jan 24;13(1):e0191404. doi: 10.1371/journal.pone.0191404 (PMC5783390; doi:10.1371/journal.pone.0191404)
Supplement: S4 Table — IQR = interquartile range; SABA = short-acting β2-agonist. *Patients may be included more than once with a different index prescription date. Number of unique patients is 7938. †Mann-Whitney. ‡Daily dose calculated as: (count of inhalers * doses in pack) / 365) * μg strength. (DOCX) [file pone.0191404.s004.docx]

|  | | **Unmatched cohorts** | | |
| --- | --- | --- | --- | --- |
|  | | **Salbutamol Comparator**  **(n=1335)** | **Salbutamol Reference**  **(n=66,736)*** | ***P*-value**  **(Chi-square)** |
| Number of prescriptions for SABA inhalers recorded in the year prior to the index prescription date | Median (IQR) | 6 (0; 15) | 7 (0; 17) | <0.001† |
| Number of baseline prescriptions for SABA inhalers, categorized, n (%) | 0 | 213 (16.0) | 9626 (14.4) | <0.001 |
|  | 1-3 | 272 (20.4) | 11,340 (17.0) |  |
|  | 4-6 | 215 (16.1) | 11,661 (17.5) |  |
|  | 7-9 | 222 (16.6) | 10,045 (15.1) |  |
|  | 10-13 | 227 (17.0) | 12,536 (18.8) |  |
|  | 14+ | 186 (13.9) | 11,528 (17.3) |  |
| Prescribed daily dose of SABA inhalers (µg)^‡^ in the year prior to the index prescription date | Median (IQR) | 438.36 (0; 1479.45) | 547.95 (0; 1753.42) | <0.001† |
| SABA inhalers daily dose (µg), categorized, n (%) | 0 | 213 (16.0) | 9626 (14.4) | <0.001 |
|  | ≤200 | 198 (14.8) | 7473 (11.2) |  |
|  | >200-400 | 215 (16.1) | 10,175 (15.2) |  |
|  | >400-600 | 174 (13.0) | 7871 (11.8) |  |
