# Supplementary material for: Real-life effectiveness and safety of salbutamol Steri-Neb™ vs. Ventolin Nebules® for exacerbations in patients with COPD: Historical cohort study
Source: PLoS One. 2018 Jan 24;13(1):e0191404. doi: 10.1371/journal.pone.0191404 (PMC5783390; doi:10.1371/journal.pone.0191404)
Supplement: S5 Table — ICS = inhaled corticosteroid; LABA = long-acting beta agonist; LAMA = long-acting muscarinic anatagonist; LTRA = leukotriene antagonist; SABA = short-acting β2-agonist; SAMA = short-acting muscarinic antagonist; THEO = theophylline. *Patients may be included more than once with a different index prescription date. Number of unique patients is 7938. †Salbutamol reference. Applies to changing (within the comparator cohort) and continuing (within the reference cohort) subcohorts only. ‡All courses that are definitely not maintenance therapy and/or all courses where dosing instructions suggest exacerbation treatment (eg, 6–1 reducing, or 30 mg as directed) and/or all courses with no dosing instructions, but unlikely to be maintenance therapy, with a code for COPD or a lower respiratory event. §Lower respiratory diagnostic codes (including asthma, COPD and lower respiratory tract infection (LRTI) read codes) or asthma/COPD review codes excluding any monitoring letter codes or lung function and/or asthma monitoring AND any additional respiratory examinations, referrals, chest x-rays, or events. (DOCX) [file pone.0191404.s005.docx]

|  | | **Unmatched cohorts** | | |
| --- | --- | --- | --- | --- |
|  | | **Salbutamol Comparator**  **(n=1335)** | **Salbutamol Reference**  **(n=66,736)*** | **P-value**  **(Chi-square)** |
| Number of patients who received at least 1 prescription for other respiratory drugs (other than SABA) in the year before index prescription date, n (%) | SABA nebulizers^†^ | 140 (10.5) | 62,515 (93.7) | <0.001 |
|  | ICS | 1004 (75.2) | 53,119 (79.6) | <0.001 |
|  | LABA | 375 (28.1) | 17,454 (26.2) | 0.111 |
|  | LAMA | 205 (15.4) | 6143 (9.2) | <0.001 |
|  | LTRA | 63 (4.7) | 2922 (4.4) | 0.547 |
|  | SAMA | 450 (33.7) | 23,613 (35.4) | 0.205 |
|  | THEO | 313 (23.4) | 24,676 (37.0) | <0.001 |
| Prescriptions for acute oral corticosteroids‡ with lower respiratory consultation^§^ in the year before index prescription date, n (%) | 0 | 754 (56.5) | 48,868 (73.2) | <0.001 |
|  | 1 | 282 (21.1) | 9648 (14.5) |  |
|  | 2+ | 299 (22.4) | 8220 (12.3) |  |
| Prescriptions for antibiotics with lower respiratory consultation in the year before index prescription date, n (%) | 0 | 646 (48.4) | 36,770 (55.1) | <0.001 |
|  | 1 | 296 (22.2) | 12,517 (18.8) |  |
|  | 2+ | 693 (29.5) | 17,449 (26.2) |  |
