# Supplementary material for: Real-life effectiveness and safety of salbutamol Steri-Neb™ vs. Ventolin Nebules® for exacerbations in patients with COPD: Historical cohort study
Source: PLoS One. 2018 Jan 24;13(1):e0191404. doi: 10.1371/journal.pone.0191404 (PMC5783390; doi:10.1371/journal.pone.0191404)
Supplement: S6 Table — ICS = inhaled corticosteroid; LABA = long-acting beta agonist; LAMA = long-acting muscarinic anatagonist; LTRA = leukotriene antagonist; SABA = short-acting β2-agonist; SAMA = short-acting muscarinic antagonist; THEO = theophylline. *Patients may be included more than once with a different index prescription date. Number of unique patients is 7938. (DOCX) [file pone.0191404.s006.docx]

|  | **Unmatched cohorts** | | |
| --- | --- | --- | --- |
|  | **Salbutamol Comparator**  **(n=1335)** | **Salbutamol Reference**  **(n=66,736)*** | ***P*-value**  **(Chi-square)** |
| NONE | 118 (8.8) | 2253 (3.4) | <0.001 |
| SABA | 100 (7.5) | 4237 (6.3) |  |
| SAMA | 11 (0.8) | 475 (0.7) |  |
| SAMA + SABA | 27 (2) | 2302 (3.4) |  |
| LABA (+/- SAMA +/- SABA) | 39 (2.9) | 1983 (3) |  |
| LAMA (+/- SAMA +/- SABA) | 10 (0.7) | 553 (0.8) |  |
| LABA + LAMA (+/- SAMA +/- SABA) | 1 (0.1) | 79 (0.1) |  |
| ICS (+/- SAMA +/- SABA) | 392 (29.4) | 27,885 (41.8) |  |
| ICS + LABA (+/- SAMA +/- SABA) | 398 (29.8) | 18,393 (27.6) |  |
| ICS + LAMA (+/- SAMA +/- SABA) | 16 (1.2) | 546 (0.8) |  |
| ICS + LABA + LAMA (+/- SAMA +/- SABA) | 155 (11.6) | 4361 (6.5) |  |
| LTRA (+/- SAMA +/- SABA) | 0 (0) | 285 (0.4) |  |
| LABA + LTRA (+/- SAMA +/- SABA) | 1 (0.1) | 309 (0.5) |  |
| LAMA + LTRA (+/- SAMA +/- SABA) | 0 (0) | 13 (0) |  |
| ICS + LTRA (+/- SAMA +/- SABA) | 9 (0.7) | 406 (0.6) |  |
| ICS + LAMA + LTRA (+/- SAMA +/- SABA) | 0 (0) | 10 (0) |  |
| ICS + LABA + LAMA + LTRA (+/- SAMA +/- SABA) | 23 (1.7) | 576 (0.9) |  |
| ICS + LABA + LTRA (+/- SAMA +/- SABA) | 27 (2) | 1304 (2) |  |
| LABA + LTRA + THEO (+/- SAMA +/- SABA) | 1 (0.1) | 19 (0) |  |
| OTHER (Theophylline only) | 7 (0.5) | 747 (1.1) |  |
