# Supplementary material for: Real-life effectiveness and safety of salbutamol Steri-Neb™ vs. Ventolin Nebules® for exacerbations in patients with COPD: Historical cohort study
Source: PLoS One. 2018 Jan 24;13(1):e0191404. doi: 10.1371/journal.pone.0191404 (PMC5783390; doi:10.1371/journal.pone.0191404)
Supplement: S7 Table — COPD = chronic obstructive pulmonary disease. *Patients may be included more than once with a different index prescription date. Number of unique patients is 7938. (DOCX) [file pone.0191404.s007.docx]

|  | | **Unmatched cohorts** | | |
| --- | --- | --- | --- | --- |
|  | | **Salbutamol Comparator**  **(n=1335)** | **Salbutamol Reference**  **(n=66,736)*** | ***P*-value**  **(Chi-square)** |
| Moderate and severe COPD exacerbations in the year prior to and including index prescription date, n (%) | 0 | 512 (38.4) | 31,603 (47.4) | <0.001 |
|  | 1 | 318 (23.8) | 14,243 (21.3) |  |
|  | 2 | 223 (16.7) | 8217 (12.3) |  |
|  | 3+ | 282 (21.1) | 12,673 (19) |  |
| Severe COPD exacerbations (hospitalizations) in the year prior to and including index prescription date,  n (%) | 0 | 1253 (93.9) | 62,387 (93.5) | 0.520 |
|  | 1+ | 82 (6.1) | 4349 (6.5) |  |
