# Supplementary material for: Real-life effectiveness and safety of salbutamol Steri-Neb™ vs. Ventolin Nebules® for exacerbations in patients with COPD: Historical cohort study
Source: PLoS One. 2018 Jan 24;13(1):e0191404. doi: 10.1371/journal.pone.0191404 (PMC5783390; doi:10.1371/journal.pone.0191404)
Supplement: S8 Table — AE = adverse event; IHD = ischemic heart disease. *Patients may be included more than once with a different index prescription date. Number of unique patients is 7938. †Includes atrial fibrillation, tachycardia, extrasystoles, and palpitations. ‡Due to small numbers chi-square results may be invalid. (DOCX) [file pone.0191404.s008.docx]

|  | | **Unmatched cohorts** | | |
| --- | --- | --- | --- | --- |
|  | | **Salbutamol Comparator**  **(n=1335)** | **Salbutamol Reference**  **(n=66,736)*** | ***P*-value**  **(Chi-square)** |
| Headache | 0, n (%) | 1292 (96.8) | 65,286 (97.8) | 0.010 |
|  | 1+, n (%) | 43 (3.2) | 1450 (2.2) |  |
| Bronchospasm/Paradoxical bronchospasm | 0, n (%) | 1304 (97.7) | 65,721 (98.5) | 0.018 |
|  | 1+, n (%) | 31 (2.3) | 1015 (1.5) |  |
| Cardiac arrhythmias^†^ | 0, n (%) | 1296 (97.1) | 65,232 (97.7) | 0.105 |
|  | 1+, n (%) | 39 (2.9) | 1504 (2.3) |  |
| Collapse | 0, n (%) | 1326 (99.3) | 66,570 (99.8) | 0.002^‡^ |
|  | 1+, n (%) | 9 (0.7) | 166 (0.2) |  |
| Mouth and throat irritation | 0, n (%) | 1282 (96) | 64,030 (95.9) | 0.876 |
|  | 1+, n (%) | 53 (4) | 2706 (4.1) |  |
| Angioedema | 0, n (%) | 1334 (99.9) | 66,677 (99.9) | 0.869 |
|  | 1+, n (%) | 1 (0.1) | 59 (0.1) |  |
| IHD | 0, n (%) | 1204 (90.2) | 61,186 (91.7) | 0.050 |
|  | 1+, n (%) | 131 (9.8) | 5550 (8.3) |  |
| Muscle cramps | 0, n (%) | 1312 (98.3) | 66,066 (99) | 0.010 |
|  | 1+, n (%) | 23 (1.7) | 670 (1) |  |
| Peripheral vasodilatation | 0, n (%) | 1333 (99.9) | 66,719 (100) | 0.007^‡^ |
|  | 1+, n (%) | 2 (0.1) | 17 (0.0) |  |
| Tremor | 0, n (%) | 1321 (99.0) | 66,489 (99.6) | <0.001 |
|  | 1+, n (%) | 14 (1.0) | 247 (0.4) |  |
| Hypokalaemia | 0, n (%) | 1334 (99.9) | 66,665 (99.9) | 0.726 |
|  | 1+, n (%) | 1 (0.1) | 71 (0.1) |  |
| Urticaria | 0, n (%) | 1332 (99.8) | 66,505 (99.7) | 0.453 |
|  | 1+, n (%) | 3 (0.2) | 231 (0.3) |  |
| Hypotension | 0, n (%) | 1335 (100) | 66,692 (99.9) | - |
|  | 1+, n (%) | 0 (0) | 44 (0.1) |  |
| Hyperactivity | 0, n (%) | 0 (0) | 0 (0) | - |
|  | 1+, n (%) | 0 (0) | 0 (0) |  |
| Lactic acidosis | 0, n (%) | 0 (0) | 0 (0) | - |
|  | 1+, n (%) | 0 (0) | 0 (0) |  |
| Any adverse event | 0, n (%) | 1048 (78.5) | 54,920 (82.3) | 0.001 |
|  | 1, n (%) | 183 (13.7) | 7764 (11.6) |  |
|  | 2+, n (%) | 104 (7.8) | 4,052 (6.1) |  |
