# Supplementary material for: Real-life effectiveness and safety of salbutamol Steri-Neb™ vs. Ventolin Nebules® for exacerbations in patients with COPD: Historical cohort study
Source: PLoS One. 2018 Jan 24;13(1):e0191404. doi: 10.1371/journal.pone.0191404 (PMC5783390; doi:10.1371/journal.pone.0191404)
Supplement: S9 Table — AE = adverse event; CLR = conditional logistical regression; IHD = ischemic heart disease. Data are expressed as number (%) of patients who had no AEs recorded or had them recorded at least once in the year prior to the index prescription date. *Includes atrial fibrillation, tachycardia, extrasystoles, and palpitations. (DOCX) [file pone.0191404.s009.docx]

|  | | **Matched cohorts** | | |
| --- | --- | --- | --- | --- |
|  | | **Salbutamol**  **Comparator**  **(n=1191)** | **Salbutamol Reference**  **(n=1191)** | ***P*-value**  **(CLR)** |
| Headache | 0, n (%) | 1151 (96.6) | 1153 (96.8) | 0.479 |
|  | 1+, n (%) | 40 (3.4) | 38 (3.2) |  |
| Bronchospasm/paradoxical bronchospasm | 0, n (%) | 1164 (97.7) | 1162 (97.6) | 0.782 |
|  | 1+, n (%) | 27 (2.3) | 29 (2.4) |  |
| Cardiac arrhythmias* | 0, n (%) | 1154 (96.9) | 1156 (97.1) | 0.806 |
|  | 1+, n (%) | 37 (3.1) | 35 (2.9) |  |
| Collapse | 0, n (%) | 1182 (99.2) | 1189 (99.8) | 0.050 |
|  | 1+, n (%) | 9 (0.8) | 2 (0.2) |  |
| Mouth and throat irritation | 0, n (%) | 1142 (95.9) | 1143 (96.0) | 0.918 |
|  | 1+, n (%) | 49 (4.1) | 48 (4.0) |  |
| Angioedema | 0, n (%) | 1190 (99.9) | 1190 (99.9) | 1.000 |
|  | 1+, n (%) | 1 (0.1) | 1 (0.1) |  |
| IHD | 0, n (%) | 1079 (90.6) | 1079 (90.6) | 1.000 |
|  | 1+, n (%) | 112 (9.4) | 112 (9.4) |  |
| Muscle cramps | 0, n (%) | 1170 (89.2) | 1175 (98.7) | 0.413 |
|  | 1+, n (%) | 21 (1.8) | 16 (1.3) |  |
| Peripheral vasodilatation | 0, n (%) | 1189 (99.8) | 1190 (99.9) | 0.571 |
|  | 1+, n (%) | 2 (0.2) | 1 (0.1) |  |
| Tremor | 0, n (%) | 1179 (99) | 1186 (99.6) | 0.100 |
|  | 1+, n (%) | 12 (1) | 5 (0.4) |  |
| Hypokalaemia | 0, n (%) | 1190 (99.9) | 1190 (99.9) | 1.000 |
|  | 1+, n (%) | 1 (0.1) | 1 (0.1) |  |
| Urticaria | 0, n (%) | 1188 (99.7) | 1183 (99.3) | 0.147 |
|  | 1+, n (%) | 3 (0.3) | 8 (0.7) |  |
| Hypotension | 0, n (%) | 1191 (100) | 1190 (99.9) | - |
|  | 1+, n (%) | 0 (0) | 1 (0.1) |  |
| Hyperactivity | 0, n (%) | 0 (0) | 0 (0) | - |
|  | 1+, n (%) | 0 (0) | 0 (0) |  |
| Lactic acidosis | 0, n (%) | 0 (0) | 0 (0) | - |
|  | 1+, n (%) | 0 (0) | 0 (0) |  |
| Any adverse event | 0, n (%) | 935 (78.5) | 943 (79.2) | 0.862 |
|  | 1, n (%) | 162 (13.6) | 151 (12.7) |  |
|  | 2+, n (%) | 94 (7.9) | 97 (8.1) |  |
